# A nature-based health intervention at a military healthcare center: a randomized, controlled, cross-over study

**DOI:** 10.7717/peerj.10519

**Published:** 2021-01-04

**Authors:** Rezvan Ameli, Perry Skeath, Preetha A. Abraham, Samin Panahi, Josh B. Kazman, Frederick Foote, Patricia A. Deuster, Niha Ahmad, Ann Berger

**Affiliations:** 1 NIMH representative to the Clinical Center, National Institutes of Health, Bethesda, MD, United States of America; 2 Pain and Palliative Care Service, National Institutions of Health, Bethesda, MD, United States of America; 3 Human Performance Partners Corp, Alexandria, VA, United States of America; 4 Consortium for Health and Military Performance, Department of Military & Emergency Medicine, F. Edward Hébert School of Medicine, Uniformed Services Universty of the Health Sciences, Bethesda, MD, United States of America; 5 Henry M. Jackson Foundation for the Advancement of Military Medicine, Bethesda, MD, United States of America; 6 Institute for Integrative Health, Baltimore, MD, United States of America

**Keywords:** Green environment, Mindfulness, Nature-based intervention, Qualitative research

## Abstract

We describe a mixed qualitative and quantitative research study in a military facility regarding the role of nature in well-being. Study intervention included two 20-minute walks. One walk was in an intentionally designed woodland environment (Green Road) and the other was on a busy campus road in a medical treatment facility (Urban Road). Twelve volunteers from a military facility participated in both walks in a cross-over experimental design. The two walking sessions were randomly ordered and preceded by pre-walk instructions appropriate to each road’s characteristics and incorporated focused attention and present moment orientation. A semi-structured post-walk interview, the primary outcome, was conducted after the conclusion of each walk. Qualitative data analyses consisted of sentiments and themes by using NVivo 12 software. The Green Road was unanimously rated as positive (100%). Responses to Urban Road were evenly distributed among positive (33.3%), negative (33.3%), and neutral/mixed (33.3%) sentiments. The Green Road yielded predominantly positive themes such as enjoyment of nature, relaxation, and feelings of privacy and safety. Urban Road produced significantly more negative themes such as concerns for safety, dislike of noise and other noxious experiences. Quantitative assessment of distress and mindfulness with Distress Thermometer (DT) and Mindful Attention Awareness Scale-state version (MAAS) demonstrated that a walk on the Green Road significantly decreased distress and increased mindfulness compared to a walk on the Urban Road. We also observed that pre-walk instructions could direct attention to both obvious and subtle elements of experience and enhance awareness. Results support the notion that an intentional nature-based environment may produce significantly more positive experiences and result in health-promoting benefits in a military health-care setting compared to an urban environment. Future studies with clinical populations could advance our understanding of the healing value of nature-based interventions. The impact of intentional green environments may be enhanced by well-designed instructions for both recreational and therapeutic use.

## Introduction

Military service members spend an inordinate amount of their lives on military installations. As a result, in the US Department of Defense at large, there is an overall commitment to use installations’ existing environments to optimize service member health and wellbeing throughout the readiness cycle (Caravalho Jr, 2015; Shams-White et al., 2019). Domestic military treatment facilities are a special instance of this, as they provide primary and specialized inpatient care to service members from all around the country. Naval Support Activity, Bethesda, home of Walter Reed National Military Medical Center (NSA/B/ Walter Reed), is the largest joint military treatment facility (WRNMMC, 2020). It includes a number of specialty treatment programs for wounded and transitioning service members, who often have complicated physical and mental ailments that require prolonged inpatient care. NSA/B expanded significantly with the 2005 closure/merger the National Naval Medical Center and Walter Reed Army Medical Center. Concurrently, efforts have been underway to leverage evidence-based design principles (Casscells, Kurmel & Ponatoski, 2009) to make NSA/B a patient-centered environment that fosters holistic healing and welcomes patients and their families (Foote, 2012; Foote et al., 2012). One relevant approach for this is to create intentional green spaces with implicit and explicit invitations to relax, reflect, and enjoy.

Complementary and ancillary interventions may enhance the success of efforts to promote better physical and mental health, and quality of life for military service members (Madsen, Vaughan & Koehlmoos, 2017). Among ancillary programs, many involve outdoor activities (Stigsdotter et al., 2011) such as sports (Caddick, Smith & Phoenix, 2014; Caddick, Smith & Phoenix, 2015; Marchand et al., 2018), horticulture (Gorman & Cacciatore, 2017; Lehmann, Detweiler & Detweiler, 2018), nature adventure (Gelkopf et al., 2013; Hyer et al., 1996), equine and other animal therapies (Fine & Beck, 2015), and spending time in nature (Poulsen, Stigsdotter & Davidsen, 2018; Poulsen, Stigsdotter & Refshage, 2015; Wagenfeld, Roy-Fisher & Mitchell, 2013). Military hospitals and other health-care facilities designed for the care of veterans and military personnel incorporate intentional healing environments that often include spending time in nature (US Department of Veterans Affairs, 2020).

City living impacts mental health negatively and contributes to increased amygdala activity, negative affect, stress, mood and anxiety disorders (Diorio, Viau & Meaney, 1993; Lederbogen et al., 2011; Peen et al., 2010) whereas exposure to green spaces has been found to be protective (Tost et al., 2019). Extensive research into green spaces and forest bathing demonstrates their stress-reducing effects and their potential to foster physical and mental healing (Bielinis et al., 2018; Doimo, Masiero & Gatto, 2020; Hansen & Jones, 2020; Kotera, Richardson & Sheffield, 2020; Park et al., 2011).

Enhancing the impact of intentional green spaces is clearly desirable. Mindfulness, which is the capacity to engage awareness in the present moment (Ameli, 2013), has been found to increase connectedness to nature (Wang et al., 2016). A meta-analysis by Schutte & Malouff, (2018) suggests that there could even be a reciprocal relationship between connectedness to nature and mindfulness.

Even though green spaces clearly have the potential to reduce stress and improve health and wellbeing, little research has been conducted to examine their effects in military installations.

The purpose of our research on the Green Road at the NSA/B was to assess participants’ subjective experience of spending time in nature by walking in an intentionally designed nature environment (Green Road), in comparison to the subjective experience of walking through a busy campus street (Urban Road) (Data S1). Our primary outcome was qualitative analysis of a semi-structured interview that inquired about participants’ subjective experiences. To the best of our knowledge, a controlled study on the effectiveness of walking in different environments in a military health care setting has not been conducted. We also developed pre-walk instructional scripts appropriate to each road’s characteristics and incorporated elements of focused attention and present moment orientation to enhance mindfulness during the walks. We hypothesized that the Green Road walk would produce more frequent positive sentiments and themes compared to the Urban Road walk. In addition, we assessed self-reported stress reduction and enhanced mindfulness (Wielgosz et al., 2019). We utilized responses to the Distress Thermometer (DT) and Mindful Attention Awareness Scale-state version (MAAS) as secondary outcomes. We hypothesized that stress would decrease, and mindfulness would increase to a greater degree after the Green Road walk compared to the Urban Road walk.

## Methods

### Study design

This is a single site, randomized, multiple methods, and cross-over design. We are reporting on qualitative findings as part of our phase one study. Self-report secondary outcomes for Distress Thermometer and Mindful Attention Awareness Scale are also reported. The study was supported by the Consortium for Health Performance (CHAMP) at Uniformed Services University (USU) and a grant awarded from TKF Foundation/Nature Sacred (NatureSacred, 2017) in collaboration with National Institutes of Health (NIH), and took place at NSA/B/Walter Reed in Bethesda, Maryland. Data were collected from September of 2018 to August of 2019. The study was approved by the USU Institutional Review Board (IRB; MEM-91-2415 [398931]). Participants signed informed consent (supplement) and met inclusion/exclusion criteria. Permission for audio recording of interviews was requested during the consent processes. At the start of the qualitative interview, participants were given control of the tape-recorder and informed that they could stop recording, end the interview, or withdraw their data and participation at any time. They were not required to answer every question. Participants served as their own control (cross-over design). The order of the two walks (Green or Urban walk) was random. Both walks were completed within one week. Participants completed a demographic questionnaire and self-report assessments before the study. They also completed self-report measures before and after each walk and participated in a semi-structured qualitative interview, the primary outcome measure, after each walk.

### Participants

Twelve (12) volunteer personnel participated in the study. They were included if they were a service member (active duty, reserve, or National Guard), military retiree, military family member, caregiver, student, or employee at the NSA/B/Walter Reed. Other inclusion criteria included being ambulatory; 18–60 years of age; able to speak and read English; able to complete study questionnaires; willing to abstain from food, alcohol and tobacco one hour prior to the experiment; have no overt heart disease, and/or any diagnosis that would limit ability to understand procedures or make informed consent such as severe Traumatic Brain Injury (TBI) or active psychosis; and for women not be pregnant or lactating. The average age was 35 years, with nine women and three men. No other gender identification was made. Table 1 provides demographic information.

### Instruments

#### A. Roads

1. The **Green Road** (the Nation’s largest wild-type healing garden) was completed in September, 2017. It is intended to provide healing and stress relief to the 12,000 military personnel at Naval Support Activity, Bethesda (NSA/B), home of the Walter Reed National Military Medical Center (WRNMMC) (NSA/B/Walter Reed), especially for those with traumatic brain injury and post-traumatic stress disorder. The two-acre garden sits in an eight-acre woodland ravine, surrounding an existing natural stream. The site connects with a $ \frac{1}{2} $ mile woodland path across the Navy Base, allowing pedestrians to avoid traffic and other stressors while moving about the Base. The woodland garden site was modified to give enhanced exposure to trees, water, stone, and wild animals (deer, aquatic life, and other woodland fauna). These were cited as desirable elements by focus groups of military personnel. A Communal and a Commemorative Pavilion were also constructed. The project was a public/private partnership between the Navy Base and the Institute for Integrative Health (Baltimore) with substantial funding from TKF Foundation (Annapolis, MD), the friends of Shockey Gillet, Capital Funding, LLC, and other donors.

**Table 1:** Sample characteristics (*n* = 12).

Age, Mean (SD)	35 (12)
Gender	
Male	3 (25%)
Female	9 (75%)
Race	
African American	3 (25%)
Asian	3 (25%)
Caucasian	4 (33%)
Other	2 (17%)
Highest Education	
High School	1 (8%)
College	5 (42%)
Graduate School	6 (50%)
Psychological Disorders	
Depression	
Yes	2 (17%)
No	10 (83%)
Substance Use	
Yes	0 (0%)
Anxiety	
Yes	2 (17%)
No	10 (83%)
Post-Traumatic Stress Disorder	
Yes	2 (17%)
No	10 (83%)
Any Mood Disorder	
Yes	4 (33%)
No	8 (67%)

**Notes.**

Values are sample sizes and percentages, unless otherwise stated.

A walk through the Green Road takes an average of 20 min, which is an acceptable span of time to reduce stress (Hunter, Gillespie & Chen, 2019).

2. The **Urban Road** consists of campus sidewalks and crosswalks along relatively busy campus streets surrounded by buildings, parking garages, wayfinding signage, small grassy areas, and occasional trees. The Urban Road borders on a memorial plaza featuring a Navy anchor in the form of a large, upright cement plaque, a tall bust statute with a dedication plaque to honor Walter Reed for his contributions, and several low walls with plaques listing Medical Honor Recipients. Compared to the Green Road, the Urban Road would be considered man-made with limited touches of nature. Data S1 shows an aerial view of the NSA/B/Walter Reed campus and the location of the two roads. The location was selected to approximate the length of the Green Road for a 20-minute walk away from and back to the research laboratory building.

#### B. Qualitative Instruments

##### 1. Pre-Walk Scripts

Guidance and instructions appropriate to the characteristics of each road were developed (Data S2). These scripts provided instructions to the participants, oriented them to the roads, and helped focus their attention on unique features of each road with an emphasis on increased awareness and present moment orientation.

##### 2. Post-Walk Semi-Structured Interview

The primary qualitative outcome measure was a semi-structured interview conducted soon after the completion of the walk on each road. It was designed to elicit participants’ sentiments and themes of their experience for both walks. Participants were not required to answer every question. The interview was preceded by written reminders to the interviewer as well as verbal instructions to the participant (Data S3). After a question about the participants’ general experience (Question 1), the interview included 10 additional questions, for a total of 11, to further elicit details of the participants’ experiences. Question 1 was the most open-ended and did not provide any direction, reminders, or topics. Question 2–9 were more specific and inquired about various domains such as thoughts, feelings, sensations, and other experiences. The interviewer was not required to ask every question. To avoid redundancy, if a question was already answered by the participant, no additional inquiries were made. Question 10 and 11 were asked to elicit any additional information that had not been previously covered by the interview questions. This gave participants another opportunity to share information. Question 10 emphasized participants’ specific likes and dislikes to bring neutrality to both states of experience in response to the two roads, and to potentially offset social desirability. The same interview was administered after each walk.

#### C. Self-Report Instruments

Secondary outcome measures included the Distress Thermometer and Mindful Awareness Attention Scale.

**1. Distress Thermometer (DT) (Visual Analog Scale)** (Roth et al., 1998) is a one-item, self-report pencil and paper measure consisting of a line or thermometer image with a 0-10 scale ranging from ‘No Distress’ at one end to ‘Extreme Distress’ at the other. Participants were asked to rate their level of distress at the moment of rating. It was administered 4 times during the study, i.e., before and after each walk.

**2. Mindful Attention Awareness Scale (MAAS), state version**, (Brown & Ryan, 2003) is a 5-item self-report with a 0–6 scale ranging from ‘Not at All’ to ‘Very Much’. It is designed to assess the short term or current expression of a core characteristic of mindfulness, namely, a receptive state of mind in which attention is directed to what is occurring in the present and observes what is taking place. The state MAAS draws items from the trait MAAS which has shown acceptable reliability (e.g., Cronbach’s alpha = 0.92) (Black et al., 2012; Brown & Ryan, 2003).

### Procedure

Volunteer personnel from NSA/B/Walter Reed who met criteria either during an in-person or phone screening were invited to participate. They signed the informed consent document prior to their participation and completed the necessary paperwork and ratings before their first experimental walk. Assessments included the pre-walk scripts, post-walk semi-structured interviews, DT, and MAAS. The order of the walks was randomized. The walks occurred on separate days and within one week. Before each walk, participants received the pre-walk script and completed the DT and MAAS. At the conclusion of each walk, the semi-structured interview, DT, and MAAS were completed. The interviews which constituted the primary qualitative outcome measure were conducted by the same experienced interviewer. De-identified digital copies of semi-structured interview audio recordings were transmitted to the NIH for qualitative data analysis (QDA).

### Statistical analysis

Statistical analysis included qualitative (QDA) and quantitative approaches. QDA was performed using NVivo 12 software package (QSR International Pty Ltd., 2020). In addition to NVivo 12, two members of the research team independently assessed, classified, and categorized responses that were ambiguous. If there were disagreements, a third team member, without knowledge of the first two raters’ categorizations, broke the tie. There were few such disagreements.

Quantitative analyses of self-report measures used the nonparametric Wilcoxon Signed Rank test to examine differences in DT ratings and MAAS scores across the two study conditions. Analyses included three comparisons: post-walk Green Road versus post-walk Urban Road ratings; pre-post changes before and after walking on the Green Road; and pre-post changes before and after walking on the Urban Road. Effects sizes for the Wilcoxon Signed Rank test were quantified using *r*, which is the Wilcoxon z-value divided by the square root of the number of observations.

## Results

### A. Qualitative

Qualitative data were analyzed in terms of sentiments and themes. Sentiments classified participants’ experiences into positive, negative, or neutral/mixed categories (Table 2). Themes further described the content of responses and explored thematic categories (Table 3). Saturation of themes was achieved by the sixth participant. QDA was continued through 12 participants to ensure new themes did not emerge. No new themes emerged from analysis of participants 7–12, which confirmed saturation. Saturation after only a small number (five to ten) is common when the interview data are rich in relevant information (Malterud, Siersma & Guassora, 2015). However, the larger number of participants provided sufficient self-report data to assess stress and mindfulness.

#### 1. Sentiments

Experiences after each of the two walks were categorized as positive, negative, or neutral/mixed (Data S4). Sentiments relating to the participants’ general experience on the Green Road (Question 1) indicated that all 12 participants (100%) had a positive experience when walking this road, whereas responses to the Urban Road experiences were varied and were evenly distributed among positive (33.3%), negative (33.3%), and neutral/mixed (33.3%) sentiments. Additional interview questions (Question 2–9) revealed more information. Most of Question 2–9 responses to the Green Road experience were also positive with few neutral/mixed experiences and no negative ones. The Urban Road experience, however, continued to produce a mixture of positive, negative, and neutral/mixed experience Question 2–8. Question 9 provided uniformly positive responses for the Green Road and predominantly positive responses for the Urban Road. Landmarks on both roads were appreciated with no negative sentiments. Question 10 and 11 probed for any additional likes, or dislikes, or other experiences that were not previously stated. Table 2 summarizes participants’ Sentiments. Since participants were not required to answer every question, the total number of responses per question varied.

### 2. Themes

A variety of themes emerged during the semi-structured post-walk interviews for both the Green and Urban Roads which, in alphabetical order, included appreciation and gratitude, enjoyment, God or higher power, landscape and architecture, nature, noxious experiences, physical experiences, present moment orientation, privacy, relaxation, rush, safety, stress, and unprompted mention of topics unique to pre-walk scripts. Green Road produced more positive and Urban Road more negative Themes. Table 3 provides information on theme categories. Consistent with qualitative research, numbers are not provided in the table since meaningful numbers could be offered only when the item is well defined, delineated and exactly sampled, which is not the case in the present qualitative research (Greenhalgh & Taylor, 1997; Maxwell, 2010). Overall, positive comments when walking the Green Road were frequent for the following themes: appreciation and gratitude; enjoyment; thoughtful and beautiful integration of nature and human-made features; and positive comments about nature. Such comments were uncommon and infrequent with respect to the Urban Road walk.

**Table 2:** Sentiments expressed by the participants during the interviews upon completion of their walks.

	Green Road			Urban Road		
Post-walk interview Question	Positive	Neutral or Mixed	Negative	Positive	Neutral or Mixed	Negative
**Q1: Overall Experience**	12	0	0	4	4	4
**Q2: Thoughts**	10	2	0	3	5	4
**Q3: Physical and Sensations**	10	2	0	4	4	4
**Q4: Emotions**	11	1	0	6	2	4
**Q5: Cognition and Memory**	11	1	0	0	5	1
**Q6: Meaning**	12	0	0	4	3	2
**Q7: Connection**	8	1	0	4	4	0
**Q8: Relationship**	12	0	0	3	7	2
**Q9: Landmarks**	11	1	0	9	3	0
**Q10: Additional Likes/ Dislikes**	5	4	1	4	4	1
**Q11: Additional Comments**	7	3	0	2	3	1

**Table 3:** Themes in Alphabetical Order.

Theme name (additional descriptors)	Green Road (GR)	Urban Road (UR)
Appreciation and Gratitude	Frequent appreciation of nature, calm, beauty, memorials, design elements, privacy	Some appreciation of memorials, time off to walk, being part of military medicine
Enjoyment (feeling happy, satisfied, content, joyful, interested, restored, invigorated, energized, entertained, more alive)	Frequent reports of visual and auditory enjoyment of woodland, birds, bird songs, squirrels, deer, vegetation, and stream of water	Infrequent comments about enjoyment, and effortful attempt to find something enjoyable
God / Higher-power	Several references to God or a higher power	Rare reference to God or a higher power
Landscape and Architecture	Frequent appreciation of thoughtful and beautiful integration of nature and human-made features, wide paved path, places to sit and reflect, memorials conveying sacredness	Some appreciation of buildings and military symbols
Nature	Frequent positive comments about nature, sun, beauty of nature, sights, sounds and smells of woods, animals, stream of water	Infrequent references to nature, effortful attempt at , for example, focusing on sky or bird sounds
Noxious experiences (irritation, impatience, annoyance, fear)	Infrequent reports of dislike for litter, unfinished construction, construction noise, mosquitos, rare reports of becoming fearful during exposure to a dog and a snake	Frequent references to dislike for lack of privacy, high noise level, safety concerns, crowdedness, lack of order and cleanliness, and some references to fatigue, low mood
Physical experiences	Frequent reports of increased physical sensations related to mental relaxation, slower pace, enjoyment of the walk, sensations of warmth, physical energy, decreased pain or discomfort such as headache or back ache	Some reports of appreciation for the health benefit of walking, feeling energized by walking
Present-moment orientation (“being in the moment,” being present, mindful, ”centered)	Frequent expressions of ability to let go of worries, staying in the moment, mindful presence	None stated
Privacy	Frequent comments about liking privacy, being away from people, feeing free, able to reflect	Frequent comments about dislike of walking in a crowded place
Relaxation	Frequent reports of feeling relaxed, slow, quiet, calm, no pressure, no rush, care-free	None reported
Rush	None reported	Frequent reports of feelings of uneasiness about busy-ness, urgency, pressure, thoughts trending toward work and responsibilities
Safety	Frequent expressions of feeling safe, free, secure	Frequent reports of concern for safety due to traffic, people
Stress (anxiety, cognitive burden, distraction, fear, worry, uncertainty)	None reported	Frequent expression of stress due to proximity of traffic, crossing streets, people, noise level, safety concerns, need for vigilance
Unprompted mention of topics that are unique to pre-walk scripts	Frequent references to stream, present moment orientation, pleasant sounds of nature, enjoyment, beauty, small animals, bridges, pavilions, paved path	Frequent references to traffic, noise, people, rushing

**Notes.**

Frequency of expressions from least to most include: none, rare, infrequent, some, several, frequent.

### B. Self-Report Results

After walking on the Green Road, levels of distress were lower and mindfulness was higher compared to the Urban Road. For both of these outcomes, the effect size could be categorized as strong (based on r) (Table 4). With regard to the individual roads, after walking on the Green Road, participants reported less distress and greater mindfulness compared to pre-walk levels. In contrast, after walking on the Urban Road, distress and mindful awareness levels did not significantly change from baseline (Table 4). Prior to walking on each road, median differences in scores can be observed, but within-subject pre-walk scores did not significantly differ across the two walks (mindfulness: *z* = 0.59, *p* = 0.55; distress: *z* = 0.65, *p* = 0.52).

**Table 4:** Comparison between post-GR and post-UR mindfulness and distress ratings by using Wilcoxon signed-rank test (*n* = 12).

	**Green road**				**Urban road**				**Post green vs post urban road**	
	**Pre**	**Post**	**ES**	***p***	**Pre**	**Post**	**ES**	***p***	**ES**	***p***
Mindful Attention Awareness Scale^a^	4.5 (3.0, 9.5)	1.5 (0.0, 4.0)	0.52	0.01	2.5 (2.0, 9.0)	3.0 (2.0, 7.8)	0.02	0.92	0.48	0.02
Distress Thermometer^b^	1.5 (1.0, 2.0)	0.0 (0.0, 0.0)	0.61	<0.01	1.0 (1.0, 2.0)	1.0 (0.0, 4.0)	0.19	0.34	0.51	0.02

**Notes.**

Values are medians and inter-quartile ranges in parentheses. Effect size, r, was computed by dividing Wilcoxon’s z by the square root of the number of observations. ES = Effect Size.

a For the Mindful Attention Awareness Scale, higher scores indicate less mindfulness. Range of possible scores is 0 to 30 (observed range: 0–16).

b For Distress Thermometer scale, range of possible scores is 0–10 (observed range: 0–5).

## Discussion

This study reports the results of a mixed methods research study in a military medical facility that examined responses to intentional natural surroundings compared to a city/urban medical environment. Qualitative analyses of semi-structured interviews (Data S3) demonstrated that walking the Green Road elicited positive experiences much more frequently and reliably than walking the Urban Road. In contrast, participants’ subjective responses to the Urban Road spanned a wide range from positive to neutral/mixed to negative. Consistent with qualitative analyses, self-report assessments of distress and mindfulness before and after the walks demonstrated that walking the Green Road decreased distress and increased mindfulness compared to walking the Urban Road.

A large number of prior studies have compared nature and urban experiences and the near universal finding among these studies is that natural green environments promote health and well-being (Choudhry et al., 2015; Hartig et al., 2014; Sandifer, Sutton-Grier & Ward, 2015; Wendelboe-Nelson, Kennedy & Cherrie, 2019). This near-universal finding supports that natural environments are generally enjoyed more than urban ones. Our data are consistent with prior findings of nature’s beneficial effects compared to urban environments (Bielinis et al., 2018; Doimo, Masiero & Gatto, 2020; Hansen & Jones, 2020; Kotera, Richardson & Sheffield, 2020; Park et al., 2011).

Our study was different from most prior studies in that we provided pre-walk scripts (Data S2) that included instructions and guidance informing the participants about the salient and some subtle elements of each walk; the scripts also suggested adoption of a mindful mental attitude before starting the walk. Qualitative data show that guiding participants’ attention and attitude towards nature affected the quality of their experience in both natural and urban environments. In the post-walk interviews, present moment orientation and increased awareness of obvious and subtle aspects of the roads, both pleasant and unpleasant, were common. Notably, present moment orientation and increased awareness of physical surroundings were incorporated in the pre-walk scripts. This suggests that pre-walk scripts had a priming effect on increasing the awareness of participants’ experiences which might have otherwise been overlooked (Doyen et al., 2012). Literature supports the connection between mindfulness and nature (Schutte & Malouff, 2018). Nonetheless, it is possible that the differences between the pre-walk scripts could have biased the participants and primed them positively towards the Green Road and negatively towards the Urban Road given we incorporated the road characteristics into the scripts.

Indeed, present moment orientation—ability to let go, become present, enjoy the unique qualities of the Green Road—emerged as a theme contributing to feeling relaxed and rejuvenated after the walk. In addition, our self-report data show that a walk on the Green Road, supported by instructions to mindfully stay present, resulted in increases in mindfulness and decreases in stress levels compared to walking on the Urban Road corroborating qualitative findings. Present moment orientation is a key component of mindfulness, and the benefits of mindfulness and its impact on stress reduction have been strongly supported by the literature (Ameli et al., 2020; Shapiro et al., 2005; Vonderlin et al., 2020). We believe that the adoption of guidance to stay present and mindful, which also captures the unique qualities and characteristics of intentional natural environments, can enhance their positive impact. Mindfulness guidance instructions could be presented as permanent signage located at all entrances of intentional nature-based environments to enhance their benefits.

Limitations of the current report include a sample size that is sufficient for qualitative data analysis but small for broader inferences. In addition, the generalizability of our data to other populations, age groups, or institutional environments requires caution. The participants of the study were self-selected and social desirability bias should be considered. In addition, our secondary outcome measures were limited in their scope and included only two short self-report measures. Furthermore, pre-walk scripts were likely impactful and it is possible that they could have biased the participants differentially towards one of the roads. Future studies should design such instructions with special care and with additional scrutiny, vigilance, and attention to details.

## Conclusion

In conclusion, the results of the present study support the notion that intentional nature-based environments compared to urban environments may enhance positive experiences and result in health promoting benefits in military health-care settings. We also observed that pre-walk instructions can direct attention to both obvious and subtle elements of experiences and can enhance awareness. We encourage future studies to pay special attention to the details, purpose, and message of such instructions. Future studies with clinical populations could advance our understanding of the healing value of nature-based interventions. The positive impact of intentional nature-based environments may be enhanced by well-designed instructions for both recreational and therapeutic use. In addition, inclusion of clinical groups, such as those exposed to trauma and stress, in studies that combine various therapeutic interventions with nature interventions to produce positive psychological change is needed.

### Authors’ note

The opinions and assertions expressed herein are those of the author(s) and do not necessarily reflect the official policy or position of the Uniformed Services University or the Department of Defense.

The contents of this publication are the sole responsibility of the author(s) and do not necessarily reflect the views, opinions or policies of The Henry M. Jackson Foundation for the Advancement of Military Medicine, Inc. Mention of trade names, commercial products, or organizations does not imply endorsement by the US Government.

##  Supplemental Information

10.7717/peerj.10519/supp-1 Supplemental Information 1 Aerial Pictures Imagery ©2019 Google, Map data ©2019 Google.

10.7717/peerj.10519/supp-2 Supplemental Information 2 Pre-walk Scripts

10.7717/peerj.10519/supp-3 Supplemental Information 3 Semi-Structured Interviews

10.7717/peerj.10519/supp-4 Supplemental Information 4 Verbatim Examples

10.7717/peerj.10519/supp-5 Supplemental Information 5 Raw Numeric Data (for Table 4)

## Additional Information and Declarations

### Data Availability

The following information was supplied regarding data availability: The raw data, including aerial pictures, pre-walk scripts, semi-structured interviews, and verbatim examples, and raw numeric data for Mindful Attention Awareness Scale and Distress Thermometer, are available in the Supplemental Files.
