# Supplementary material for: A nature-based health intervention at a military healthcare center: a randomized, controlled, cross-over study
Source: PeerJ. 2021 Jan 4;9:e10519. doi: 10.7717/peerj.10519 (PMC7789867; doi:10.7717/peerj.10519)
Supplement: Supplemental Information 1 — Imagery ©2019 Google, Map data ©2019 Google. [file peerj-09-10519-s001.docx]

**Supplemental Data 1.**

**Arial View of Green and Urban roads**


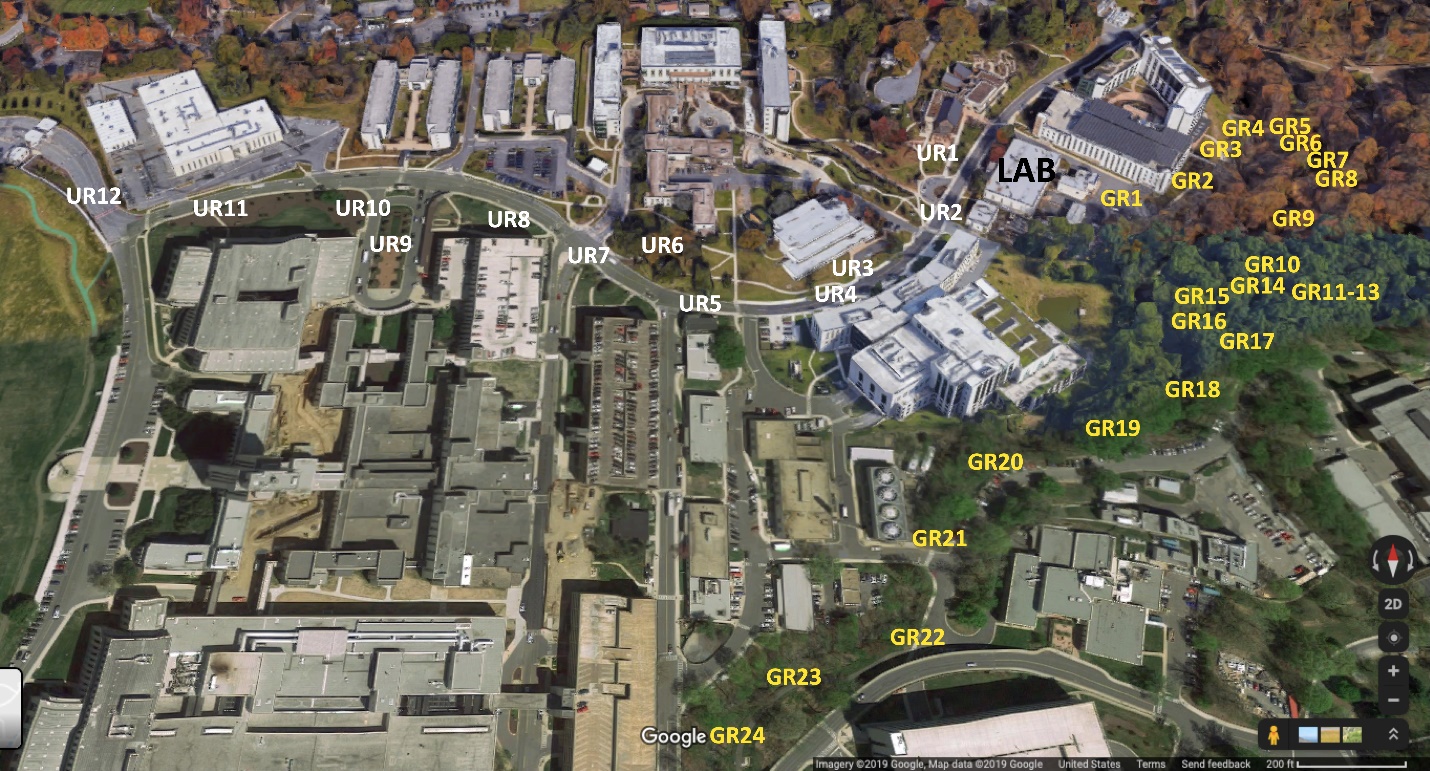


**Ground View of Green Road**


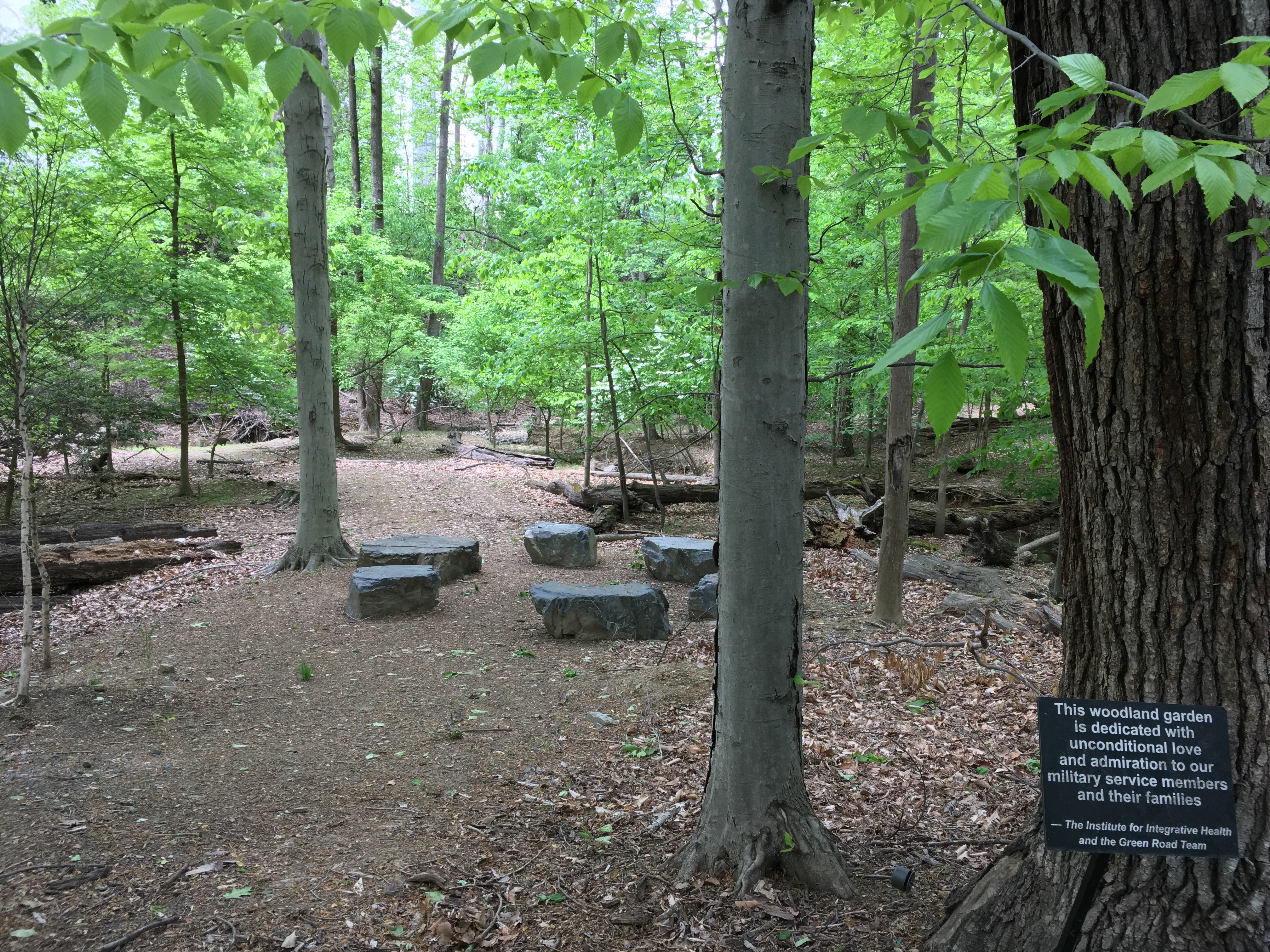


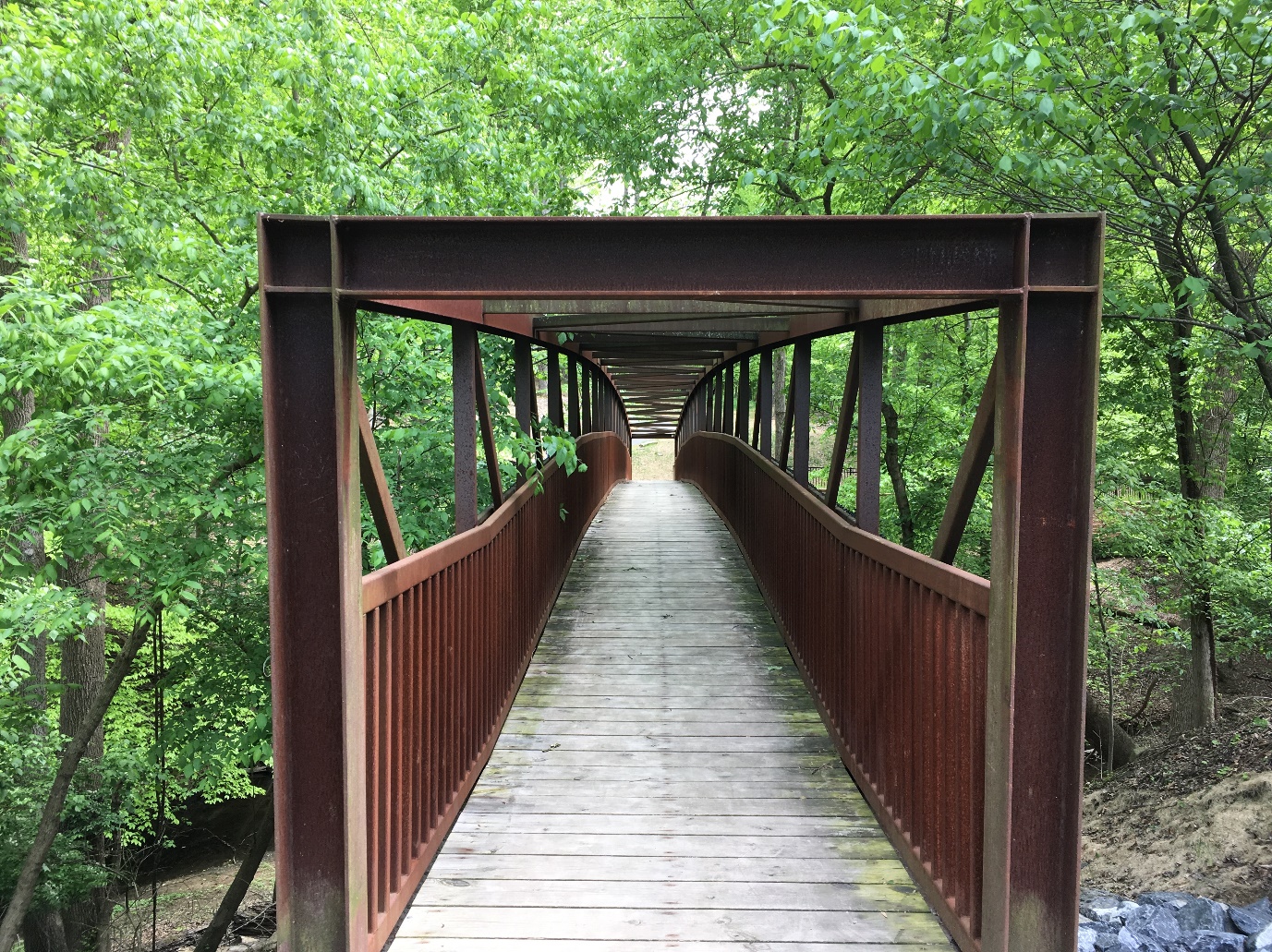


Photos were taken by the research team.

**Ground View of Urban Road**


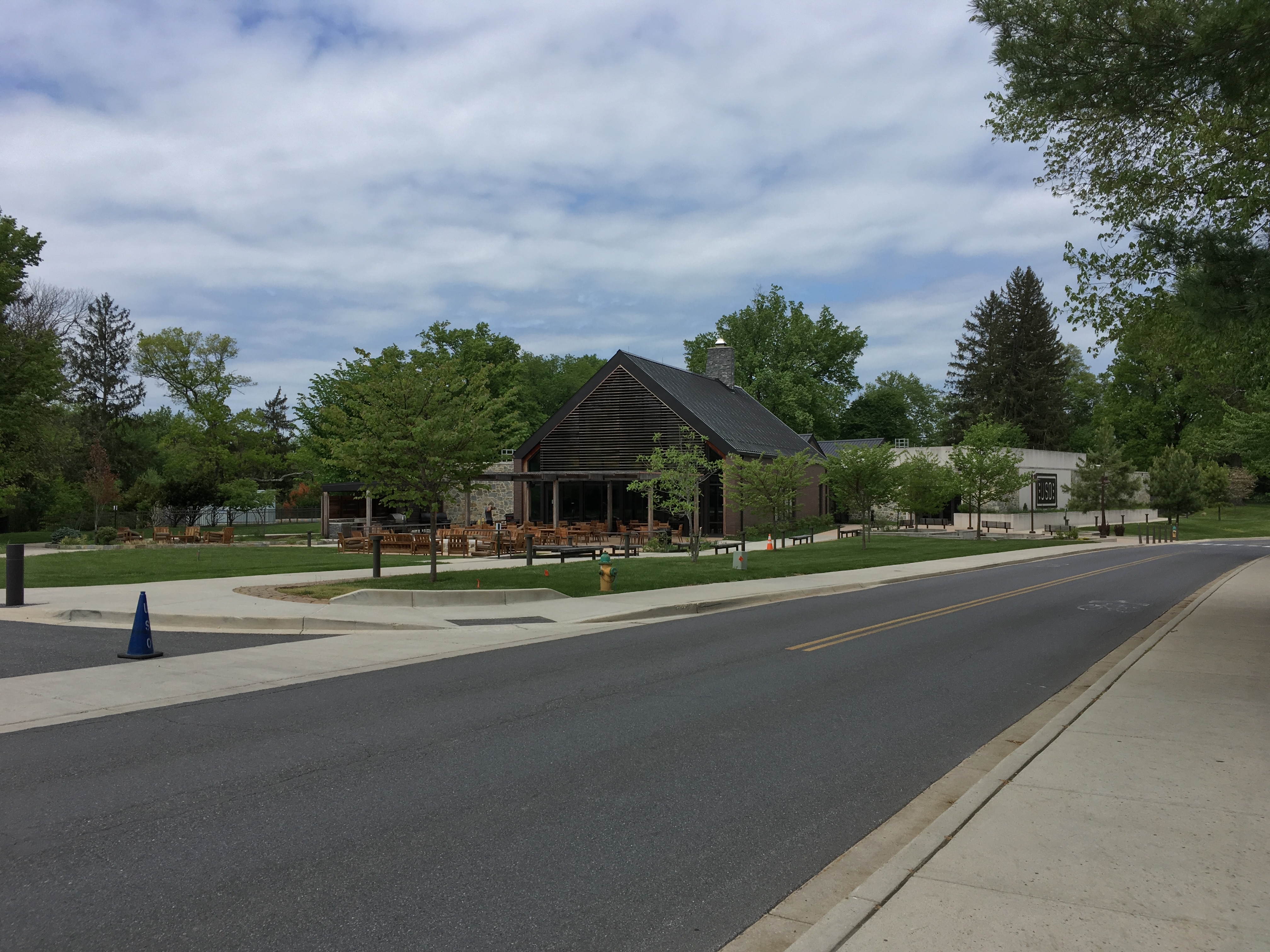

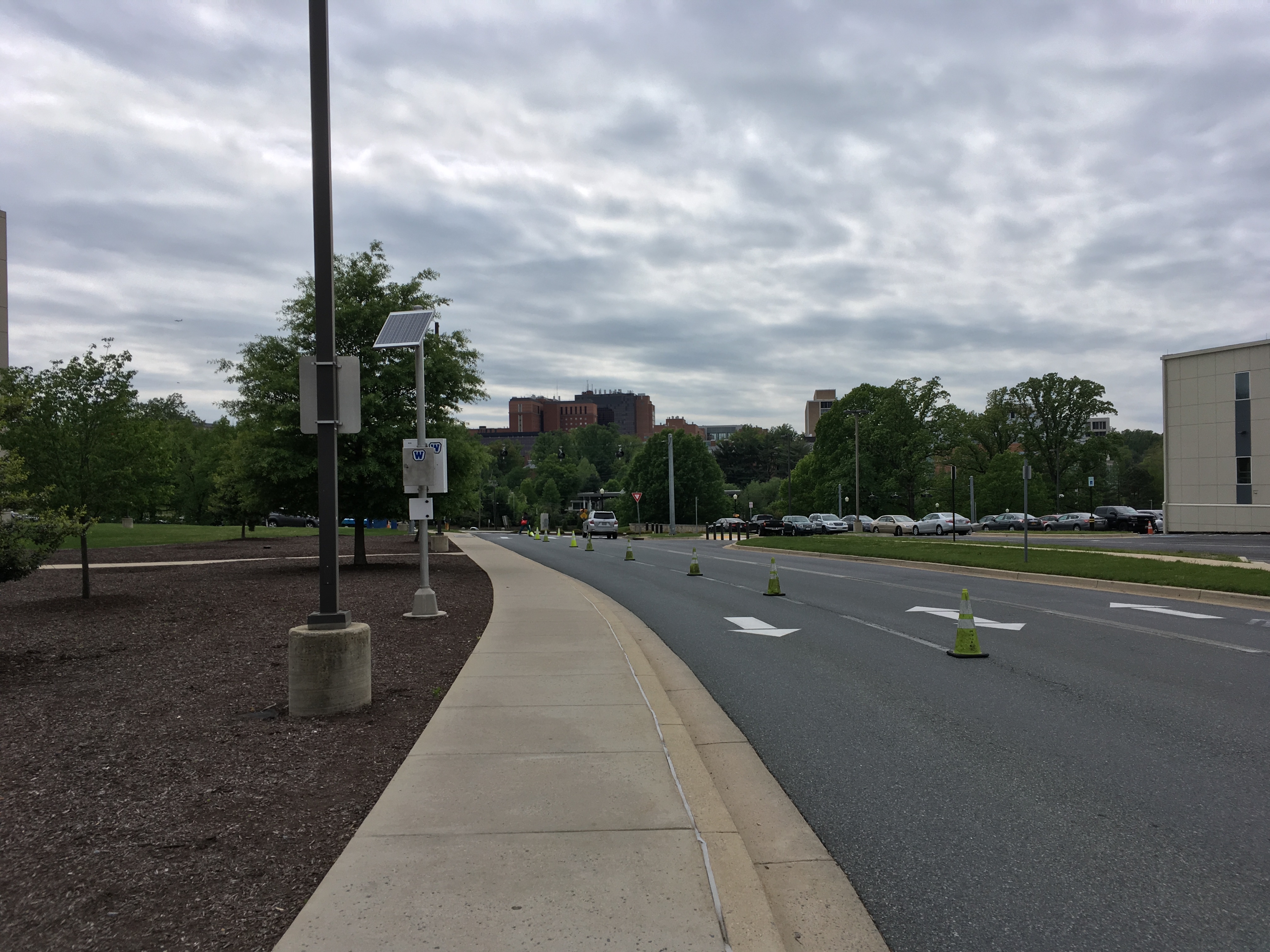


Photos were taken by the research team.
