# Supplementary material for: A nature-based health intervention at a military healthcare center: a randomized, controlled, cross-over study
Source: PeerJ. 2021 Jan 4;9:e10519. doi: 10.7717/peerj.10519 (PMC7789867; doi:10.7717/peerj.10519)
Supplement: Supplemental Information 2 [file peerj-09-10519-s002.docx]

**Supplemental Data 2. Pre-Walk Scripts**

**Urban Road Pre-Walk Script**

You are entering an open area where there are traffic and people. You will walk straight up this path all the way to the hospital close to the North Gate. There will be no right or left turns but a straight road.

As you walk, you will notice several buildings that are office spaces for the employees who work here. You may also come across several police officers, uniformed service members, civilians, or perhaps even people you know.

You may hear the noise of construction or fire trucks and may see some traffic or road signs along the path. You may also feel the sun shining on you as well as notice the trees on the side. Try to keep an open mind and do not restrict any thoughts that come your way. Again, experience these sensations and continue the walk.

If you pass any benches, it is okay if you want to sit down and rest for a few minutes to gather your thoughts and view your surroundings.

You are encouraged to walk for at least 20 minutes and explore the path and observe your surrounding areas. We have set a timer that will ring at the end of 10 minutes. At this time, you will slowly prepare to complete the walk and come back to this location, which is where we initially started. One of our research staff will be here at all times should you need anything.

Do you have any questions?

**Green Road Pre-Walk Script**

You are entering the Green Road woodland area. There is a path for you to follow as shown in the map here. As you can see, there are trees surrounding the path as you enter the woods. The path is well-kept, and on the sides, you will see small plants and soil.

You will come across certain man-built structures. These structures are considered to be sacred places to honor lost comrades and loved ones. Feel free to spend some reflective time in these areas. There are also various benches along the path for you to rest and reflect.

You will also pass a small stream. You may hear the rippling sounds of the water along with the birds chirping and the breeze fluttering through the leaves on the trees. Enjoy these sounds.

You may also come across some animals that reside in this habitat such as deer, squirrels, ants, butterflies, bees, etc. Continue to walk as you enjoy and admire these sights.

You are encouraged to walk for at least 20 minutes and explore the path and surrounding areas. In order for you to enjoy your time with nature, we have set a timer that will ring at the end of 10 minutes. At this time, you will slowly prepare to complete the walk and come back to this location, which is where we initially started. One of our research staff will be here at all times should you need anything.

Do you have any questions?
