# Supplementary material for: A nature-based health intervention at a military healthcare center: a randomized, controlled, cross-over study
Source: PeerJ. 2021 Jan 4;9:e10519. doi: 10.7717/peerj.10519 (PMC7789867; doi:10.7717/peerj.10519)
Supplement: Supplemental Information 3 [file peerj-09-10519-s003.docx]

**Supplemental Data 3. Semi-Structured Interview**

**Instructions to the Interviewer**

1. Be aware of your own personal opinions about the two roads. Ensure that your feelings and ideas about the roads do not affect your questions and body language. Conduct the interview in an identical and even-handed manner regardless of which road, the Green Road or the Urban road, you are discussing with the participant.
2. Explain the purpose of the questionnaire/interview to the participant. You should read the participant instructions out loud. Make sure participants comprehend the purpose of the study, why the information they provide is important, and their rights as participants.
3. Make sure participants understand the interview questions and what is asked of them.
4. Ask the supplemental questions provided in parentheses as needed to elicit detailed responses.
5. If the participant provides ample detailed information on a topic early in the interview (e.g., about why they enjoyed their walk), there is no need to ask them to repeat the information at a later point in the interview (e.g., question 10A).

**Instructions for Participants (read to participants)**

Thank you for participating in our study. Your feedback is very important to us.

We would like to learn as much as possible about your experience during your walk, even your subtle experiences. This information will help us understand the value of walking in different outdoor environments.

We will ask you a number of questions to help us understand your experiences. Some questions are general and some are more specific. The more we learn about the details of your experience, the more we will be able to use the information to guide us in the future planning and use of our resources. You are not required to answer any questions, and you may choose to discontinue this interview at any time for any reason.

We would like to audio record all the questions and responses during this interview so that we don’t miss anything important. Is that OK?

Do you have any questions before we begin?

1. Tell us in detail about your **experience** during your walk today.

(Optional probes: What was it like? What did you notice?)

1. What were your **general thoughts** when walking through the road?

(Optional probes: What thoughts went through your mind? Did you have one kind of thought or different ones? Like what? Did your thoughts become faster or did they become slower as you walked? Did you notice any changes in the quality, flow, or quantity of your thoughts between the time you started the walk and finished the walk? If there was a change in aspects of your thoughts, did you consider it positive, negative, or neutral?)

1. What did you experience **physically**, e.g., pain, fatigue, increased energy, changes in the breath or breathing, various bodily sensations [such as tingling, heat, cold], or restlessness? What walking speed did you maintain during the walk? Did your speed change during the walk? If there were changes, what was the reason?

(Optional probes: What physical sensations did you experience? What changed? What was that like? Can you tell us more? Even if your experiences are subtle, please share them with us. Did you notice any changes physically between the time you started the walk and finished the walk? If there was a change, did you consider it positive, negative, or neutral?)

1. What did you experience **emotionally**, e.g. anxiety, sadness, fear, irritability, frustration, boredom, light-heartedness, happiness, joy, restfulness, calmness, serenity?

(Optional probes: What emotions did you experience? What was that like? Can you tell us more? Even if your experiences are subtle, please share them with us. Did you notice any changes in your emotions between the time you started the walk and finished the walk? If there was a change, did you consider it positive, negative, or neutral?)

1. Did the walk have an effect on your **attention or concentration**?

(Optional probes: What did you notice? Did you become more focused and attentive? What did you pay attention to? Did you feel more distracted? What did your attention go to? Did your attention become more expansive? What was that like? Can you tell us more? Did you notice any changes in the quality of your attention and concentration during the walk? If there was a change, did you consider it positive, negative, or neutral?)

- 1. Any memories come up? (Optional probes: What kind of memories?)

1. Did you find the walk **meaningful** in anyway?

(Optional probes: Can you tell us more? What was meaningful for you?)

1. Did the walk help you **connect** with the nature, art, or urban life in any way?

(Optional probes: Can you tell us more? What aspects of nature? What art form/s? What aspects of urban life? Describe your pleasant, unpleasant, or even neutral experiences.)

1. During the walk, did you **think about or reflect** on yourself, someone else, important relationships, or even a higher power?

(Optional probes: Can you tell us more? What were your reflections? What other reflections, if any, did you have? )

- 1. During the walk, did you **think about or reflect** on yourself?
  2. During the walk, did you **think about or reflect** on someone else or important relationships?
  3. During the walk, did you **think about or reflect** on a higher power?

1. Were there any particular landmarks during the walk that you noticed?
2. Did you enjoy or dislike the walk in any way?

(Optional probes: What made it pleasant/unpleasant/difficult?)

- 1. Did you enjoy the walk in any way?
  2. Did you dislike the walk in any way?

1. Is there anything else you would like to add and share with us? Did anything surprise you in any way? Anything else that will help us understand you experience more fully?
