# Supplementary material for: A nature-based health intervention at a military healthcare center: a randomized, controlled, cross-over study
Source: PeerJ. 2021 Jan 4;9:e10519. doi: 10.7717/peerj.10519 (PMC7789867; doi:10.7717/peerj.10519)
Supplement: Supplemental Information 4 [file peerj-09-10519-s004.docx]

**Supplemental Data 4. Verbatim examples of responses to Green and Urban Roads demonstrating positive, negative and neutral/mixed responses to the roads.**

| Question/Road | Positive | Neutral/Mixed | Negative |
| --- | --- | --- | --- |
| Q1  Green Road | … Very peaceful & relaxing, especially stream and other woodland sounds… | N/A | N/A |
| Q1  Urban Road | … Looking at buildings and architecture. Looking at new and old buildings. I found it interesting… | … I could hear like sounds of cars, but I was trying to ignore them and just focus on the bird sounds… | ...Without ear bud or any music to relax, it (Urban Road) is really stressful… |
| Q2  Green Road | … Enjoyed scenery, it held my interest, was able to be in the moment… | … Way-finding was nerve-racking at first, then really enjoyed all the details along it… | N/A |
| Q2  Urban Road | … Reflected on Walter Reed, being part of something big and meaningful, caring for wounded… | ... Little stuff I saw as I went along… | … Cars stressed me out, people are looking at me, sun was annoying… |
| Q3  Green Road | … At peace and more relaxed both mentally and physically, able to slow my walk… | … I found myself kind of wandering off the path a little bit and really slowing down, which is unusual, uh, unusual for me… | N/A |
| Q3  Urban Road | … Felt fine, enjoyed sun, didn’t rush, body was fine, no sweat… | … Walked fast, no body sensations… | … I was noticing soreness, my aches as I'm walking back, because I'm a little bit sore on my calves… |
| Q4  Green Road | … Old trees, how small I am in universe, how God loves me, laid out stress, feel refreshed and restored for sure… | … Calm, gratitude, mild joy, satisfaction, but construction was annoying at one point… | N/A |
| Q4  Urban Road | … I would say pleasure and enjoyment. Freedom as well… | … Neutral, not happy or sad… | … I was definitely irritated and at some point, sad, because, um, it wasn’t, it wasn’t a meaningful walk for me… |
| Q5  Green Road | … I was really more centered on the walk, and sometimes my mind wanders a lot, but not today. Was enjoying the walk… | … My mind was wandering. My mind went from listening to the birds, the trees, to where it was greener… | N/A |
| Q5  Urban Road | N/A | …My concentration was not based on one thing and it had to be multi---varied. I had to concentrate on the cars, on just walking… | … It's just focus on the destination… And so, I'm not looking at the trees or the ground or whatever… |
| Q6  Green Road | … Meaningful walk, calming, tension releasing, beautiful road, appreciating the peace, gave a new idea for my life to start the day with a walk… | N/A | N/A |
| Q6  Urban Road | … Meaningful, because it calmed me after, got more steps, fresh air… | … Like a fitness walk, not meaningful can be beneficial.… | … It didn’t feel like that much. It was kind of just noise, essentially white noise and I didn’t really pay attention to anything, because there wasn’t anything interesting… |
| Q7  Green Road | … The sounds. And then I still tried to look at the picture of the trees and I enjoyed and appreciated like how old they are and how tall they are. I was looking at the roots of the trees and where the water goes by because somehow, they are exposed. I thought that was beautiful… | … I think there was a lot of construction that you could hear. But at the same time, like even like the birds and the squirrels that I connected with… | N/A |
| Q7  Urban Road | … So when I'm walking in an urban setting, I feel like there's more to entertain me. My mind wanders at the fabulous things, the people, I like looking at the cars. I just enjoy that kind of interaction… | … Obviously, it's an urban environment, so there's not as much vegetation. But, you can tell that they try to make sure there was still grass and tress here and there, And there were still birds… | N/A |
| Q8  Green Road | … Surprisingly I was not ruminating, focusing on the past or future. I really got lost in the moment which is not easy for my personality. I just let go and let my feet take me… | N/A | N/A |
| Q8  Urban Road | … I was more paying attention on what's going on around me. I was more aware of what's going on right now, what's going on here… | … I think I only connected with people, but as far as surroundings, not so much because it's buildings and concretes… | … I may have thought of a stressful event or just an annoying circumstance. And I think thinking about it during the walk that was rushed made it more of an annoying circumstance… |
| Q 9  Green Road | … Streams always equates to calm and flow and serenity, I loved the little bridges, that little pavilion… | … I noticed a dog and all the landmarks and the bridges. One of the bridges had a lot of water on the ground and stuff. It was wet… | N/A |
| Q 9  Urban Road | … That little plaza stood out, noticed was cherry tree blossoms, the horseshoe area… | … I made the walk before, I tried cross to a different side of the street just to make it a little bit different. I think I was more focused on the destination and not so much paying attention to my surroundings… | N/A |
| Q10  Green Road | … I enjoyed it. It's good to get away from work and a busy life and just, I don't know, have a peace of mind to yourself… | … The only thing is just the construction, which I know nobody has any control over. But otherwise, no, it was / it was a great walk… | … I certainly didn’t expect to see a snake… |
| Q10  Urban Road | … Just listening to the birds. That was it. That was really nice. And they all had different melodies, and I started noticing that as the walk progressed. So my concentration did increase… | … I generally enjoy walking, but this was just an average walk, I was walking from point A to point B to get something to do for work… | … It was very noisy and, um, um, the fact that I was walking very slow and, um, and um, uh, I was not in a hurry, but all the people passing me by, they were in a hurry… |
| Q11  Green Road | … It was a great experience and, it's one of those things that 20 minutes can seem like an awful long time, but it was perfect! It was the perfect amount of time… I really enjoyed it… | … It surprised me how quiet it is. Like with all the construction around, it's actually really quiet back there… | N/A |
| Q11  Urban Road | … No I just think it was a nice day. It's a nice thing. For a person like me, it really makes me realize, okay, I need to slow down in life and just enjoy the simple things, basically… I really enjoyed myself… I was kind of tense and tired…after 20 minutes… I was very calm and very content… | … The only thing I wonder if like maybe it's different at different times of the day, depending on the traffic pattern. Like there wasn’t that many people out, so it was kind of chill. But if I would feel different if there were like a rush of people… | … It was not a pleasant walk. And not only because of the weather, but because it is too hot, but also because of the environment…. |
